# High-Frequency Ultrasound Assessment of Skin and Oral Mucosa in Metabolic Syndrome Patients—A Cross-Sectional Study

**DOI:** 10.3390/jcm10194461

**Published:** 2021-09-28

**Authors:** Anida Maria Băbțan, Ștefan Cristian Vesa, Bianca Adina Boșca, Maria Crișan, Carmen Mihaela Mihu, Mihaela Felicia Băciuț, Cristian Dinu, Bogdan Crișan, Radu Septimiu Câmpian, Claudia Nicoleta Feurdean, Anca Ionel, Artur Bezugly, Ioana Roxana Bordea, Aranka Ilea

**Affiliations:** 1 Oral Rehabilitation Department, Faculty of Dentistry, “Iuliu Haţieganu” University of Medicine and Pharmacy Cluj-Napoca, Babeș Street No. 15, 400012 Cluj-Napoca, Cluj County, Romania; anidamaria.babtan@gmail.com (A.M.B.); rcampian@ymail.com (R.S.C.); cbraitoru@yahoo.com (C.N.F.); anca_ionel@yahoo.com (A.I.); arankailea@yahoo.com (A.I.); 2 Pharmacology, Toxicology and Clinical Pharmacology Department, Faculty of Medicine, “Iuliu Haţieganu” University of Medicine and Pharmacy Cluj-Napoca, Marinescu Street No. 23, 400337 Cluj-Napoca, Cluj County, Romania; 3 Histology Department, Faculty of Medicine, “Iuliu Haţieganu” University of Medicine and Pharmacy Cluj-Napoca, Pasteur Street No. 4, 400349 Cluj-Napoca, Cluj County, Romania; biancabosca@yahoo.com (B.A.B.); mcrisan7@yahoo.com (M.C.); carmenmihu2004@yahoo.com (C.M.M.); 4 Maxillofacial Surgery and Implantology Department, Faculty of Dentistry, “Iuliu Haţieganu” University of Medicine and Pharmacy Cluj-Napoca, Cardinal Iuliu Hossu Street No. 37, 400029 Cluj-Napoca, Cluj County, Romania; mbaciut@umfcluj.ro (M.F.B.); dinu_christian@yahoo.com (C.D.); crisan.bogdan@umfcluj.ro (B.C.); 5 Dermatology and Cosmetology Department, Academy of Postgraduate Education of the Russian Federal Medical-Biological Agency, 123098 Moscow, Russia; drarturbezugly@gmail.com; 6 Oral Health Department, Faculty of Dentistry, “Iuliu Haţieganu” University of Medicine and Pharmacy Cluj-Napoca, Babeș Street No. 15, 400012 Cluj-Napoca, Cluj County, Romania; roxana.bordea@ymail.com

**Keywords:** high-frequency ultrasound, oral mucosa, skin, metabolic syndrome, inflammation, aging

## Abstract

Background: Exogenous factors (such as sun exposure, smoking habits, and diet) and endogenous (inflammatory status, general diseases) have a direct influence on skin and soft tissue characteristics. The study’s objective was to assess the impact of metabolic syndrome (MS) on characteristics of skin layers in sun-exposed and non-exposed maxillofacial tissues evaluated by high-frequency ultrasound (HFU), as a potential diagnosis and monitoring tool for the aging process. Material and methods: The present study included 102 subjects (24 with MS; 78 without MS). Anthropometric parameters and disease history were recorded, and blood samples were harvested in order to assess biochemical parameters of MS. Sun-exposed skin (zygomatic region) and non-exposed oral mucosa of the lower lip were assessed using HFU (DUB^®^ cutis, Taberna Pro Medicum) with a 22 MHz probe. Results: Patients with cardiac disease had significantly lower values for epidermis density (*p* = 0.002). Gender was independently linked to the aged dermis depth (*p* < 0.001), aged dermis no. of px (pixels) (*p* < 0.001), dermis depth (*p* < 0.001), dermis no. of px (*p* < 0.001), and subcutaneous tissue density (*p* < 0.001). Patients with MS had thinner epidermis (*p* = 0.008) and thinner aged dermis (*p* = 0.037) when compared to non-MS subjects. Conclusion: Patients with MS had thinner epidermis and a lower epidermis number of pixels in sun-exposed skin. Women had lower epidermis density and thicker dermis in sun-exposed skin. Our study showed that HFU, as a non-invasive investigation approach, is useful to diagnose and monitor the aging process in skin and oral mucosa, correlated with skin phenotype pathological conditions.

## 1. Introduction

Aging is a physiological process that develops in the entire human body, leading to senescence, the decline in biological features, and the adapting capacities to metabolic stress. These theories combine both psychosocial and health habits. The aging process leads to a decrease in fibroblast number and the insufficient production of type I and III collagen fibers, resulting in the collapse of the extracellular matrix, reduced skin elasticity, increased vascular fragility, and tissue vulnerability to mechanical forces [1]. Glycation is considered to be one of the main determining factors in aging, due to the generation of collateral reactive oxygen species (ROS); ROS and glycation induce cross-link bridges in collagen fiber and lead to morphological and mechanical changes, represented by fiber shortening, stiffness, frailty, and low regeneration potential [2,3]. Skin chronic exposure to ultraviolet (UV) rays is clinically evidenced by dermatologic examination, and it is expressed by tissue pigmentation and constitution of wrinkles (mainly due to loss of elasticity); histopathological investigation shows a degenerative process represented by elastosis, tissue deposition of Advanced Glycation End Products (AGEs) and glycosaminoglycans, and altered collagen synthesis [4,5]. Collagen glycation can be evidenced and quantified by high-frequency ultrasonography (HFU) due to the fluorescence of the affected structures [6].

According to the World Health Organization, metabolic syndrome (MS) is defined by the presence of diabetes mellitus or glycaemia dysregulation, associated with high blood pressure, high plasma triglycerides (TG)/low plasma high-density lipoproteins (HDL), and obesity or microalbuminuria [7,8]. It is known that patients diagnosed with MS have a disrupted dermis, due to alteration of collagen fiber structure, that can be assessed using ultrasound examination; diffuse and impaired collagen matrix is responsible for skin frailty and low mechanical strength [9]. Ultrasound applications in the maxillofacial area include investigation of lymph nodes, salivary glands, and benign and malignant tumors and monitoring of soft tissue healing, inflammation and degenerative processes, and anatomy and physiology of dento-periodontal dynamics [10,11]. HFU finds its applications in the diagnosis of pigmented lesions, autoimmune and degenerative diseases, and benign and malignant tumors [12], but also in the monitoring and controlling of the aging process [13], tissue healing, and regenerative processes [14,15,16]. HFU was previously used in MS patients in assessing vascular structure modification and remodeling processes [17].

Exogenous factors (such as sun exposure, smoking habits, diet) and endogenous factors (inflammatory status, general diseases) have a direct influence on skin and soft tissue characteristics. The study’s objective was to assess the impact of metabolic syndrome (MS) on characteristics of skin layers in sun-exposed and non-exposed maxillofacial tissues evaluated by high-frequency ultrasound (HFU) as a potential diagnosis and monitoring tool for the aging process. In order to evaluate the impact of sun exposure on tissue characteristics, sun-exposed (left zygomatic skin) and non-exposed (lower lip mucosa) maxillofacial tissues will be compared.

## 2. Materials and Methods

The present study is an analytical, observational, transversal type. The patients (*N* = 102) were recruited from Oral Rehabilitation and Prosthodontic Departments (Faculty of Dentistry) and from the Regional Diabetes Center in Cluj-Napoca, Romania, between 2018 and 2019. Inclusion criteria were patients over 18 years old who required oral cavity clinical examination and diagnosis, associated or not with dental treatment requirements and clinical status of the patients was healthy or with associated general pathologies (high blood pressure, ischemic heart disease, obesity, lipid metabolism impairment). Exclusion criteria were patients weighing more than 150 kg (which exceeded the measuring capacity of our device) and patients with skin and/or oral mucosa lesions or vascular abnormalities. The present study was approved by the University Ethical Board, no 93/08.03.2017. Written and informed consent was obtained from all subjects, according to the World Medical Association Declaration of Helsinki, revised in 2000, Edinburgh.

### 2.1. Study Protocol

Patients were enrolled at 7:30 a.m. at the Oral Rehabilitation Department and were asked to fill in the written informed consent regarding the procedures that will follow after inclusion in the clinical study. Demographic, clinical, and anthropometric measurements were recorded: age, gender, BMI, waist circumference, and skin phototype. Venous blood was harvested from the upper arm and collected in sterile osmotic tubes. MS was defined according to the NCEP/ATP III criteria (diagnosis was made when three or more are present): waist circumference of more than 102 cm in men or more than 88 cm in women, fasting triglyceride level of 150 mg/dL or higher, blood pressure level of 130/85 mm Hg or higher, high-density lipoprotein cholesterol (HDL-C) level of less than 40 mg/dL in men or less than 50 mg/dL in women, and fasting glucose level of 100 mg/dL or higher. The following parameters were measured from the blood samples: blood count, erythrocyte sedimentation rate (ESR), glycaemia (fasting blood sugar value), and lipidic profile. Regarding the diseases associated with MS, the following pathological conditions were recorded: arterial hypertension, ischemic cardiac diseases, diabetes mellitus (type I or II), and dyslipidemia.

### 2.2. Ultrasonographic Evaluation

**Figure 1:**
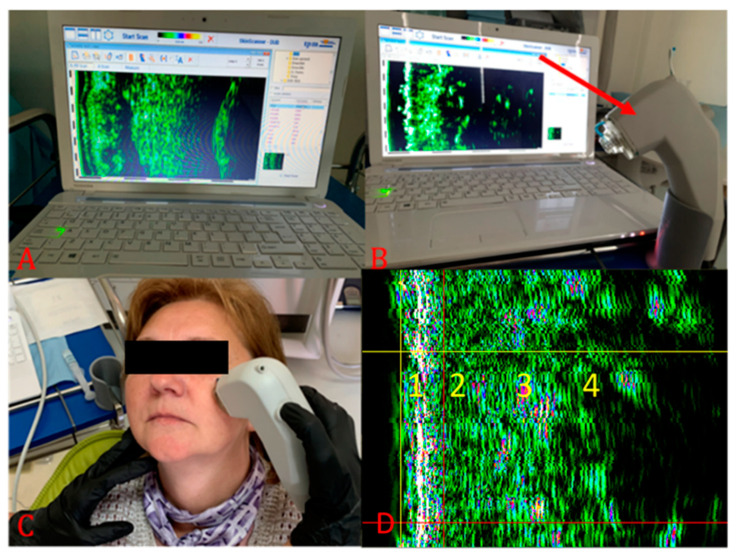
HFU assessment in zygomatic skin area. (**A**) HFU (DUB cutis, Taberna Pro Medicum, Lüneburg, Germany); (**B**) the transducer with a 22 MhZ frequency (red arrow); (**C**) assessment of the ecographic measurements; (**D**) HFU skin structures: 1—epidermis, 2—aged dermis, 3—healthy dermis, 4—hypodermis.

**Figure 2:**
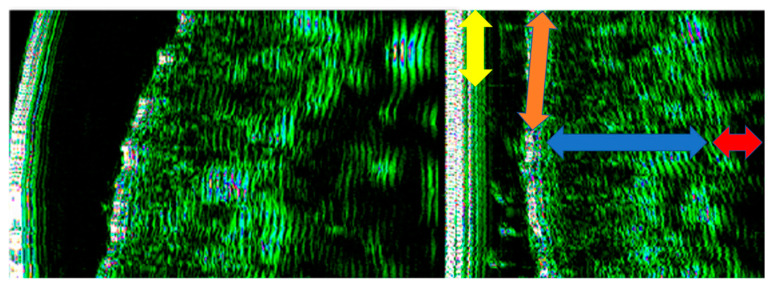
HFU (DUB cutis, Taberna Pro Medicum, Lüneburg, Germany) 22 MHz zygomatic skin evaluation. The images are exported in pixels in order to evidence tissue density (white and blue—very dense, green—dense, and black—low density). Yellow arrow—probe membrane and applied gel, red arrow—hyperpixeled epidermis; blue arrow—pixeled dermis, red arrow—low-density hypodermis.

**Figure 3:**
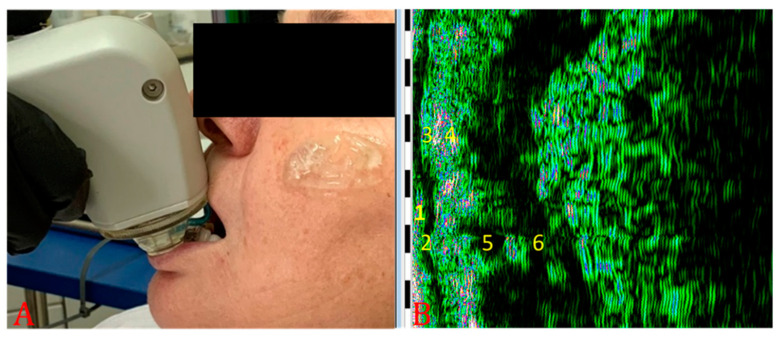
Lower lip HFU examination. (**A**) Transducer position on inner surface of the lower lip, (**B**) the lower lip ultrasound structure: 1—superficial layer of the nonkeratinized epithelium, 2—spinosum and basal layers of the nonkeratinized epithelium, 3—papillary and reticular layers of the lamina propriae, 4—submucosa, 5—minor salivary gland, 6—orbicular oris muscle.

**Figure 4:**
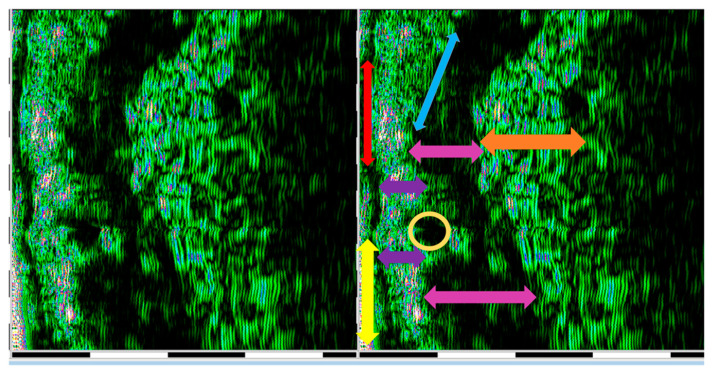
HFU (DUB cutis, Taberna Pro Medicum, Lüneburg, Germany) 22 MHz zygomatic skin evaluation: red arrow—superficial layer of the nonkeratinized epithelium, yellow arrow—spinosum and basal layers of the nonkeratinized epithelium, purple arrow—papillary and reticular layers of the lamina propriae, blue arrow—thin submucosa, pink arrow—orbicular oris muscle, yellow circle—minor salivary gland, orange arrow—conjunctiva–vascular axis.

Tissue glycation related to collagen fiber glycation reaction was indirectly assessed by an HFU device, 22 MHz (DUB cutis, Taberna Pro Medicum, Lüneburg, Germany) (Figure 1A). The depth of signal penetration was 8 mm, axial resolution 57 µm at 22 MHz. The measurements were performed with the windows covered by curtains (to avoid assessment errors induced by the UV), at a 25 °C environment temperature. The ultrasound probe (Figure 1B) was used as a transmission medium for both ultrasonographical gel (applied on the examined surface) and water introduced in the HFU probe and covered with a thin transparent membrane. The ultrasonographical images were purchased in B-scan and A-scan viewing modes. The evaluation was performed on sun-exposed skin (left zygomatic area) and non-sun-exposed tissue (inner surface of the lower lip). Before the measurements were performed, the patients were asked to remove facial moisturizer/concealer, if any was present. The transducer was arranged parallel to and without pressure both on the zygomatic and inner lower lip region, after the gel application (Figure 1C). The ultrasonography was performed by two different operators (AMB and SCV). Each measurement was carried three times by each operator. For each examined situs, the HFU was performed in three different points (mesial, central, and distal). The final value was the sum of the values. When values differences were identified, the respective measurement was resumed by the two operators, and inter-agreement between examiners was achieved. Intra-rater reliability was assessed for measurements of epidermis depth. The intraclass correlation coefficient was 0.787, which indicates good reliability. Inter-rater reliability was assessed for measurements of epidermis depth, using Cohen’s kappa that revealed a k of 0.881. For the sun-exposed skin in the zygomatic area, including the epidermis, the dermis, and the subcutaneous tissue (hypodermis), the tissue’s depth (thickness), pixels (px) count, and density (automatically) were registered (Figure 1D and Figure 2). The examined non-sun-exposed tissue was represented by the oral mucosa on the inner surface of the lower lip (Figure 3A), including the nonkeratinized epithelium, the lamina propria, and the submucosa. The following parameters were recorded: depth (thickness), pixels (px) count, and density (Figure 3B and Figure 4).

### 2.3. Statistical Analysis

Statistical analysis was performed using the MedCalc Statistical Software version 19.2.1 (MedCalc Software Ltd., Ostend, Belgium; https://www.medcalc.org; 2020). Quantitative variables were tested for normality of distribution using the Shapiro–Wilk test and were expressed as median and 25–75 percentiles. Qualitative data were characterized by frequency and percentage. Comparisons between groups were performed using the Mann–Whitney test and Kruskal–Wallis test or chi-square test, whenever appropriate. Correlations between variables were carried out using Spearman’s rank coefficient. Multiple linear regressions were employed in order to establish the independent influence of several variables on ultrasound measurements. The *p* value <0.05 was considered statistically significant.

## 3. Results

Demographic, anthropometric, and biochemical profile results are presented in Table 1. Age, gender, smoking status, and Fitzpatrick skin phenotype did not differ between groups. Patients with MS were more likely to have ischemic disease, besides the criteria for MS.

The ultrasound assessment of zygomatic skin in MS patients vs. controls is summarized in Table 2. Patients with cardiac disease had significantly lower values for epidermis density (56.6 (34.4; 74) vs. 65.4 (51.1; 82.9), *p* = 0.002). Patients with MS (Table 2) had lower epidermis depth (*p* = 0.008), epidermis no. of pixels (*p* = 0.041), and aged dermis depth (*p* = 0.037) compared to non-MS patients. Smokers had higher values of depth of aged dermis (609 (430; 744) vs. 494 (398; 602); *p* = 0.04), no. of pixels for aged dermis (9717 (7320; 11,932) vs. 8160 (6307; 9516), *p* = 0.01), and no. of pixels for healthy dermis (25,531 (21,508; 29,040) vs. 22,288 (20,233.5; 26,013), *p* = 0.01). Smokers had lower values for density of dermis (7.53 (5.5; 11.4), *p* = 0.01)) and density of healthy dermis (15.7 (10.5; 20.4) vs. 20.9 (14.7; 25.9), *p* = 0.05)) compared to non-smokers.

There were no differences regarding the ultrasound assessment of zygomatic skin between the criteria of metabolic syndrome, aside from low HDL cholesterol, which was associated with thinner epidermis depth (281(248.5; 321.2) vs. 309 (273; 344), *p* = 0.01). Patients with arterial hypertension had thinner epidermis depth, but the statistical significance threshold was slightly passed (*p* = 0.09).

**Figure 5:**
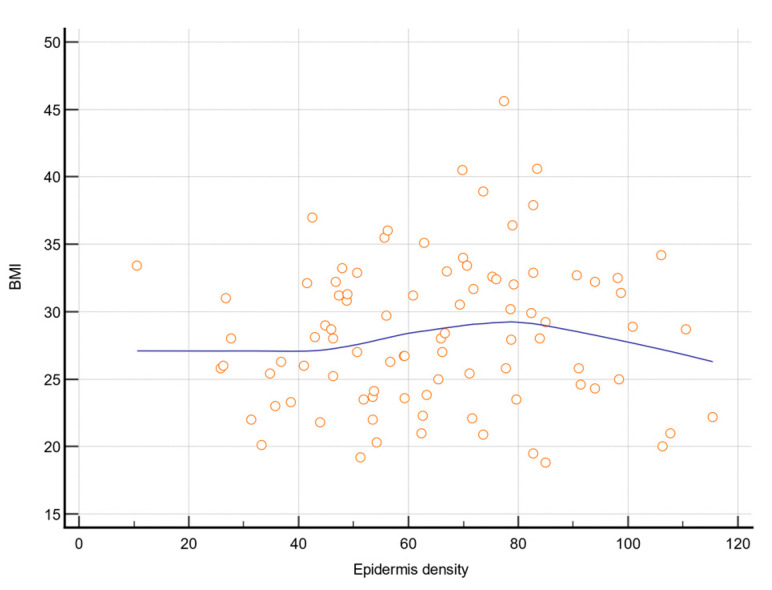
Correlation between BMI and epidermis density. BMI—Body Mass Index (kg/m^2^).

BMI significantly correlated with the epidermis density (r = 0.200; *p* = 0.005; Figure 5). Female subjects had lower epidermis density compared to male subjects (56 (43.5; 74.2) versus 68 (52.4; 77.6); *p* = 0.033) and higher subcutaneous density (11.4 (7; 16.8) versus 6.9 (4.9; 10.1); *p* = 0.003).

**Figure 6:**
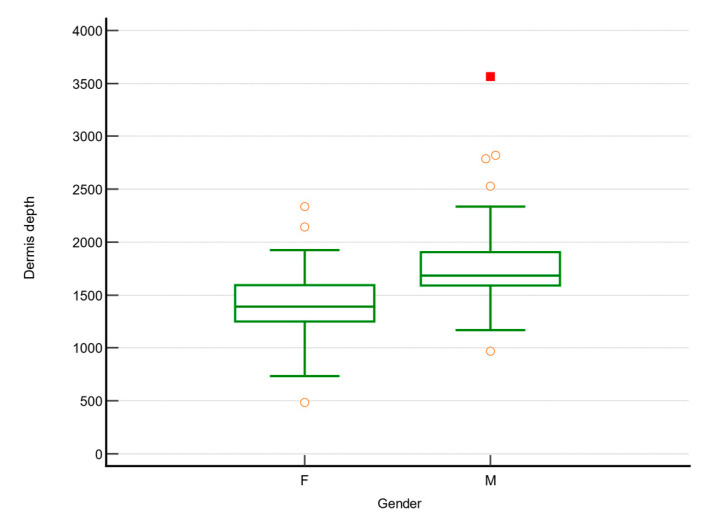
Dermis depth in females and males.

Females had thicker aged dermis (527 (412; 609) versus 719 (619.2; 1083.7); *p* < 0.001), lower aged dermis no. of pixels (8343 (6337.5; 9598.5) versus 11,845.5 (9444; 16,325); *p* < 0.001), lower entire dermis depth (1391 (1253; 1582) versus 1796 (1670; 2384.75); *p* < 0.001), Figure 6, and lower pixels count of the dermis (21,569 (19,894; 24,412.75) versus 28,047 (25,607; 37,343.75); *p* < 0.001).

When correlating the skin phenotype with the ultrasound measurements, patients with type IV phenotype had thicker dermis (*p* = 0.013) and a higher no. of pixels in the dermis (*p* = 0.012) compared to type II or III phenotypes. At the non-sun-exposed sites (lower lip mucosa), there was a significant difference regarding the depth of non-keratinized epithelium (*p* = 0.008) and pixel count (*p* = 0.035) in patients with type IV phenotype compared to types II and III (Table 3).

The number of pixels in the hypodermis of the inferior lip was significantly higher in patients with MS (26,367 (22,944; 33,387) vs. 23,504 (19,912; 28,504); *p* = 0.03). There were no significant differences between patients with MS and controls regarding the other measurements performed on the lower lip mucosa.

Several multiple linear regressions were used in order to better understand the relationship between variables and the ultrasound measurements of the zygomatic skin. We took into account the age, gender, phenotype, history of cardiac disease (other than those included in the MS definition), and the presence of MS. MS was the only variable independently associated with epidermis depth (*p* = 0.01) and epidermis no. of px (*p* = 0.03). Several variables were independently associated with epidermis density: MS (*p* = 0.03), gender (*p* = 0.01), and history of cardiac disease (*p* = 0.001). Gender was independently linked to the aged dermis depth (*p* < 0.001), aged dermis no. of px (*p* < 0.001), dermis depth (*p* < 0.001), dermis no. of px (*p* < 0.001), and subcutaneous tissue density (*p* < 0.001).

## 4. Discussion

To the best of our knowledge, this is the first study to use HFU in order to correlate the ultrasound parameters of maxillofacial tissues with the patient’s health status, specifically MS.

HFU examination assists the in vivo anatomy and physiology study of human skin. In our study, epidermis depth showed a 293 µm (266; 336) median at the zygomatic skin epidermis depth, which differs from other studies, which assessed only stratum corneum thickness using Raman spectroscopy, which obtained in the cheek region values ranging from 16 to 25 µm [18,19]. Oeslen et al. found up to 61 µm of stratum corneum in healthy patients using Reflectance Confocal Microscopy [20] and Bridal et al. 160 µm using 19–50 MHz [21]. The differences in their results are due to the individual assessment of one skin layer, compared to entire epidermis, in our study.

Aged dermis density in MS subjects for the zygomatic area was lower compared to controls, which is in accordance with the results obtained by Crișan et al., who used a similar device (Dermascan C, Cortex Technology, Hadsund, Denmark) [1,22]. Firooz et al. used the same device and found proximate values when evaluating cheek and neck dermis [23]. The difference in tissue density is given by the dehydration and the disruption of the collagen fiber extracellular matrix, which caused the loss of elasticity, wrinkle formation, and the decrease in the tissue density [24]. Tissue depths of the hypodermis and submucosa were similar in both examined areas, with a difference regarding their density. Similar values were reported by other ultrasonographical studies using the same MHz, but at different localizations, such as the erector spinae and upper abdomen [25,26]. Kim et al. used a 16 MHz ultrasound probe and found between 2.91 and 3.67 mm subcutaneous thickness in the forehead and infraorbital region [27]. The difference found in the fat tissue density might be explained by the fact that adipose tissue density ranges from 0.925 to 0.970 g/mL and is influenced by its main components (lipids, water, and dry fat-free substances) [28]. Moreover, the tissue density was associated with the adipocytes’ dimension—a higher density corresponded to smaller cells [29]. Although we anticipated that BMI would correlate with the epidermis density (r = 0.200; *p* = 0.005), we found no correlation with the subcutaneous tissue parameters.

In our study, patients with cardiac diseases had lower density of epidermis, aged dermis, subcutaneous tissue, lower no. of pixels for the inferior lip epithelium and lamina propria, and lower depth of papillary and reticular lamina propria. We found no similar studies in the literature, but it is known that in heart failure, high blood pressure, and atherosclerosis, the main pathophysiologic mechanism is the proinflammatory one, in which IL-1, IL-6, IL-8, IL-10, TGF-β, and TNF-α enhance fibroblast synthesis activity, induce cellular remodeling and the dissolution of extracellular matrix and collagen fibers, promoting a chronic degenerative-inflammatory health status. These cells enhance AGEs production, which stimulate lipid peroxidation, ROS, and the maintenance of the vicious cycle [30,31]. The systemic chronical meta-inflammation is evidenced at the tissue level, as well, by the altered structure and decreased density, the reason for which the ultrasound modification could reflect the general health status. We consider that the presence of metabolic disease led to structural and mechanical changes specific to aged tissue: decreased thickness, disrupted collagen synthesis, and loss of conjunctive tissue elasticity. The device that we used in this study allowed us to assess these modifications, knowing the fact that a healthy epidermis and dermis are characterized by increased thickness, white-dense green hyperpixeled aspect, and increased density.

Patients with MS showed a decrease in epidermis depth, no. of pixels, and aged dermis depth compared to non-MS patients. Our results are consistent with literature data stating that the epidermis has a lower thickness in subjects with MS due to the general proinflammatory status and a decrease in skin neurostimulation and hydration [32,33]. These results could help in early diagnosis of skin modification in MS patients and preventing skin deterioration by implementing regenerative and antioxidative general assays.

Patients with type IV skin phenotype had a thicker dermis and higher dermis no. of pixels compared to type II and III phenotypes, as was shown in Table 3. The hyperpixeled zygomatic skin and lower lip mucosa areas are both related to the quantity of melanine and continuous synthesis of fibroblasts. Colorimetric and spectrometric methods had been used to assess skin chromophores (melanin and hemoglobin) quantity in different skin phenotypes [34,35]. Patients with darker skin had larger, more numerous, and more dispersed melanosomes, compared to those with light skin, which contributed to the epidermis thickness and protection of the dermis, which had higher elasticity and resistance to senescence [36], results that could explain the ultrasound differences that we found between skin phototypes. At the non-sun-exposed sites, we found statistically significant differences between non-keratinized epithelium depth and no. of pixels in patients with type IV phenotype compared to types II and III. This is the first literature study of this kind; we found no similar articles.

The strength of the study was represented by the use of an HFU device (DUB cutis, Taberna Pro Medicum) with a 22 MHz probe, which aided the visualization of all skin and mucosa structures due to the depth of signal penetration. Additionally, the US evaluation of lower lip structures has not been described in other literature studies, to the best of our knowledge. We consider that this approach would contribute to early diagnosis of mucosa modifications, which could entail speeding up the diagnosis of tumor-related modifications in patients with MS and other tissue age and general disease-related issues. The comparison between HFU findings and associated disease could be a new method in paraclinical diagnosis of metabolic diseases and aging-related disease.

The limitations were related to the reduced number of patients with associated diseases, the small number of subjects with type II skin phenotype, and the lack of histopathological validation of the presented data.

## 5. Conclusions

Patients with MS had thinner epidermis and a lower epidermis number of pixels in sun-exposed skin. Women had lower epidermis density and thicker dermis in sun-exposed skin. Our study showed that HFU, as a non-invasive investigation approach, is useful to diagnose and monitor the aging process in skin and oral mucosa, correlated with skin phenotype pathological conditions.

## Figures and Tables

**Table 1:** Demographic, anthropometric, and health status characterization of the included patients with and without MS.

Characteristic		Without MS (*N* = 78)	MS (*N* = 24)	*p*
Age		49 (36; 60)	52 (42; 59)	0.8 *
Gender	Male	22 (28.2%)	10 (41.7%)	0.3 **
	Female	56 (71.8%)	14 (58.3%)	
BMI		26.9 (23.6; 31.4)	29 (22.6; 32)	0.01 *
HDL (mg/dL)		52 (41.3; 64.7)	45.1 (40.1; 48.2)	0.04 *
TC (mg/dL)		178.2 (147.7; 208.1)	190.8 (163.1; 255.9)	0.2 *
Tg (mg/dL)		122.6 (81.5; 170.1)	160.1 (130.4; 190.5)	0.001 *
LDL (mg/dL)		119.8 (89.3; 146.1)	164 (127.5; 217.1)	0.02 *
Ischemic Cardiac Diseases		3 (3.8%)	5 (20.8%)	0.006 **
Arterial Hypertension		6 (7.7%)	18 (75%)	<0.001 **
Diabetes mellitus		1 (1.3%)	5 (5.9%)	0.003 **
Hypo-HDL		26 (33.3%)	20 (83.3%)	<0.001 **
Hyper-TG		15 (19.2%)	18 (75%)	<0.001 **
Abdominal obesity		30 (38.5%)	19 (79,2%)	0.001 **
Fitzpatrick skin phenotype	2	4 (5.1%)	-	0.4 **
	3	55 (70.5%)	19 (79.2%)	
	4	19 (24.4%)	5 (20.8%)	
Smoker		41 (52.6%)	8 (33.3%)	0.1 **

Legend: *N*—number of subjects, BMI—Body Mass Index (kg/m^2^), HDL—High-Density Lipoprotein, Hypo-HDL—Hypo High Density Lipoproteinemia, TC—Total Cholesterol, Tg—Triglycerides; Hyper-Tg—Hypertriglyceridemia, LDL—Low-Density Lipoprotein, MS—Metabolic Syndrome, * Mann–Whitney test, ** chi-square test.

**Table 2:** Ultrasound assessment of zygomatic skin in MS patients vs. controls.

Variable	Non-MS Patients (*N* = 78)	MS (*N* = 24)	*p*
Epidermis depth (µm)	289 (266; 334)	266 (251.5; 299)	0.008
Epidermis no. of px	4657 (4173; 5228)	4182 (4034.5; 4864)	0.041
Epidermis density	60.01 (43.68; 78.32)	53.41 (46.68; 72.89)	0.813
Aged dermis depth (µm)	605.5 (497.5; 738.25)	523.5 (367; 648.75)	0.037
Aged dermis no. of px	9475 (8057.25; 11,572.75)	7941 (5814.75; 10,915.5)	0.091
Aged dermis density	12.43 (9.169; 15.86)	12.375 (7.035; 21.485)	0.328
Dermis depth (µm)	1586 (1341.75; 1711)	1375 (1300.75; 1759.5)	0.731
Dermis no. of px	24,185 (20,896; 26,433.75)	21,415 (20,519.75; 27,670.5)	0.485
Dermis density	17.95 (13.52; 24.54)	16.3 (14; 28.95)	0.944
Subcutaneous tissue depth (µm)	1332 (999.75; 1894.25)	1396 (1040.75; 1681.75)	0.444
Subcutaneous tissue no. of pixels	20,935.5 (15,903.25; 29,461.5)	22,245 (16,279.5; 26,468)	0.711
Subcutaneous tissue density	8.1 (5.3; 13.2)	6.8 (5.2; 10.4)	0.3

px: pixels.

**Table 3:** HFU assessment of lower lip mucosa according to skin phenotype.

	Type 2	Type 3	Type 4	*p*
Nonker. mucosa depth	586 (404; 781.5)	352 (305; 422)	336 (273; 375)	0.008
Nonker. mucosa no of px	7373 (6263.5; 11,085)	5880 (5104; 6966)	5658 (4284; 6324)	0.035
Nonker. mucosa density	12.745 (4.605; 28.992)	23.07 (14.8; 36.76)	21.86 (16.33; 47.22)	0.213
Lamina propriae depth	1129 (859.25; 1949.25)	828 (625; 1023)	875 (594; 1234)	0.142
Lamina propriae no of px	18,019.5 (13,855.5; 29,681.25)	12,980 (9628; 15.776)	16,214 (9933; 18,802)	0.084
Lamina propriae density	21.805 (15.627; 39.937)	39.49 (29.08; 54.77)	47.09 (28.26; 58.84)	0.122
Lip hypodermis depth	1129 (859.25; 1949.25)	828 (625; 1023)	875 (594; 1234)	0.636
Inf. Lip hypodermis no of px	5.315 (3.817; 9.917)	11.93 (9.20; 20.68)	12.45 (6.69; 31.18)	0.846

## Data Availability

The data presented in this study are openly available in MendeleyData at doi:10.17632/xddms253pr.1.
